# Management of Symptomatic Sacral Perineural Cysts

**DOI:** 10.1371/journal.pone.0039958

**Published:** 2012-06-29

**Authors:** Jianqiang Xu, Yongdong Sun, Xin Huang, Wenzhong Luan

**Affiliations:** 1 Department of Neurosurgery, General Hospital of Fengfeng Group, Handan, China; 2 Department of Neurosurgery, Peking University People’s Hospital, Beijing, China; University of California Los Angeles, United States of America

## Abstract

**Background:**

There has been no consensus on the optimal treatment of symptomatic sacral perineural cysts. Most previous reports concerning the management methods were either sporadic case reports or a series of limited cases. This study is to further optimize the management for patients with symptomatic sacral perineural cysts by analyzing the outcomes of a cohort of patients who were treated with different strategies.

**Methods and Findings:**

We reviewed the outcomes of 15 patients with symptomatic sacral perineural cysts who were managed by three different modalities from 1998 through 2010. Six patients underwent microsurgical cyst fenestration and cyst wall imbrication. Seven patients underwent a modified surgical procedure, during which the cerebrospinal fluid leak aperture was located and repaired. Two patients were treated with medication and physical therapy. Outcomes of the patients were assessed by following up (13 months to 10 years). All of the six patients treated with microsurgical cyst fenestration and cyst wall imbrication experienced complete or substantial relief of their preoperative symptoms. However, the symptoms of one patient reappeared eight months after the operation. Another patient experienced a postoperative cerebrospinal fluid leakage. Six of the seven patients treated with the modified surgical operation experienced complete or substantial resolution of their preoperative symptoms, with only one patient who experienced temporary worsening of his preoperative urine incontinence, which disappeared gradually one month later. No new postoperative neurological deficits, no cerebrospinal fluid leaks and no recurrence were observed in the seven patients. The symptoms of the two patients treated with conservative measures aggravated with time.

**Conclusions:**

Microsurgical operation should be a treatment consideration in patients with symptomatic sacral perineural cysts. Furthermore, the surgical procedure with partial cyst removal and aperture repair for prevention of cerebrospinal fluid leakage seemed to be more simple and effective.

## Introduction

Sacral perineural cysts, which were also termed Tarlov cysts, are collections of cerebrospinal fluid (CSF) between the endoneurium and perineurium of the nerve root sheath near the dorsal root ganglion [1]. These lesions are quite common as an incidental finding on magnetic resonance imaging (MRI), and most of them are asymptomatic [2]. In a series of 500 consecutive MRI scans of the lumbosacral spine, Paulsen et al [3] recorded an incidence of 4.6%, of which 20% were symptomatic. Approximately 1% of sacral perineural cysts become large and cause symptoms related to local compression [3], which should be treated.

There has been no consensus on the optimal treatment of symptomatic sacral perineural cysts since it was first described by Tarlov in 1938 [4]. Many methods have been applied to treat these symptomatic lesions, with variable results. Lumbar CSF drainage, lumboperitoneal shunt, and cyst subarachnoid shunt were not effective as a therapy for symptomatic Tarlov cysts [2], [5], [6]. CT-guided percutaneous aspiration of the cyst with infusion of fibrin glue yielded mixed results, and this method was associated with a high rate of aseptic meningitis [3], [7], [8]. Favorable results have been obtained with microsurgical cyst fenestration and imbrication in several reports [2], [4], [9], [10].However, there remains some difficulties such as postoperative CSF leakage and the cyst recurrence [2]–[9].

Most previous reports concerning the management methods were either sporadic case reports or series of limited cases [2], [4], [5], [10]–[14]. We therefore retrospectively reviewed 15 cases of sacral perineural cysts treated with different methods.

## Methods

Between 1998 and 2010, 15 patients (nine men, six women) ranging from 23 to 60 years of age (mean, 37.8 yr) with symptomatic sacral perineural cysts were treated at General Hospital of Fengfeng Group (Table 1),whose follow-up is more than one year. The main symptoms and neurological deficits included low back pain or sacrococcygodynia (n = 12), sacral radiculopathy (n = 7), numbness (n = 6), sensory disturbance of the sacral dermatome (n = 9), claudication (n = 4), and bowel and bladder dysfunction (n = 6). Written informed consent was obtained from each patient. The study was approved by the Medical Ethical Committee of Peking University.

**Table 1:** Summary of included patients.

Patient No.	Sex/age (yr)	Main symptoms	Cyst location	Additional pathologicalentity	Surgery	Outcome	Postoperative CSFleakage	Follow-upperiod(m)
1	F/32	local numbness	S3–S4	no	PCWRI	substantial relief	no	36
2	M/49	radicular pain	S1	no	PCWRI	substantial relief	no	18
3	M/52	bladder dysfunction	S2–S3	no	PCWRI	recurrence	no	13
4	M/23	local pain	S3–S4	no	PCWRI	complete remission	no	26
5	M/39	local pain	S2	no	PCWRI	complete remission	no	73
6	M/46	radicular pain	S2–S3	no	PCWRI	complete remission	yes	124
7	M/28	local pain	S1	no	PCRAR	complete remission	no	24
8	F/57	radicular pain	S2–S3	tethered spinal cord and intramedullary teratoma	PCRAR	complete remission	no	16
9	M/40	local pain	S1–S2 2cysts	no	PCRAR	complete remission	no	14
10	M/59	local pain	S2	no	PCRAR	complete remission	no	28
11	M/23	radicular pain	S2–S4	tethered spinal cord	PCRAR	substantial relief	no	34
12	F/26	local pain	S2	no	PCRAR	substantial relief	no	22
13	F/28	bladder dysfunction	S1–S2	no	PCRAR	complete remission	no	61
14	F/36	local pain	S1–S2 2cysts(bilateral)	no	CT	aggravated		50
15	F/29	local pain	S2	no	CT	aggravated		62

**Abbreviations:** PCWRI, partial cyst wall resection and imbrication; PCRAR, partial cyst wall resection and aperture repair; CSF, cerebral spinal fluid.

**Figure 1:**
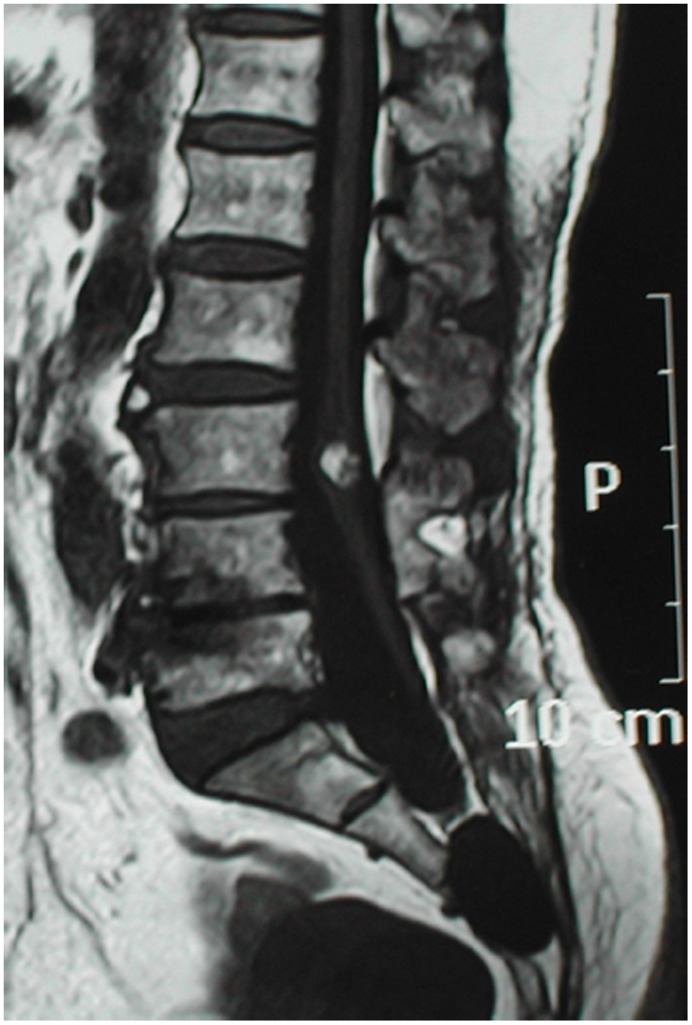
MRI findings of a cyst in the sacral spinal canal, a low-lying tethered cord and a intramedullary tumor at L3 level.

**Figure 2:**
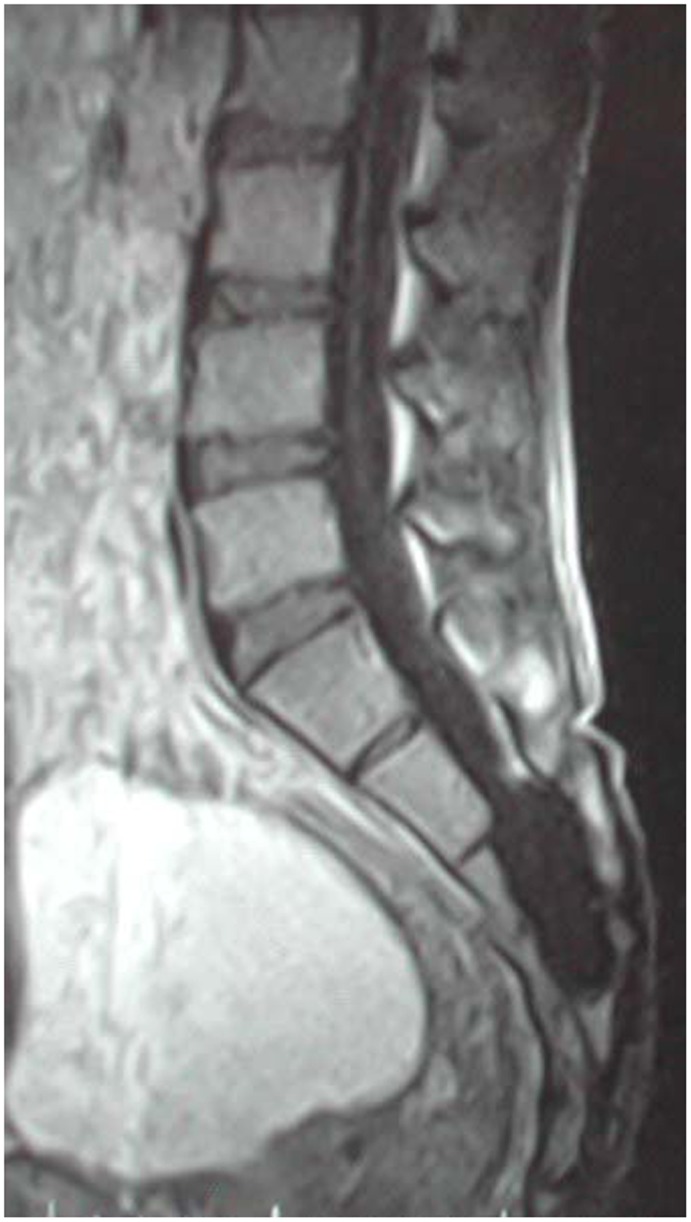
MRI findings of a cyst in the sacral spinal canal and low-lying tethered cord with syringomyelia.

The diagnoses of sacral perineural cysts were confirmed for all patients by magnetic resonance imaging (MRI) studies. Besides the cysts, MRI also demonstrated additional pathological features in two cases. Tethered spinal cord and intramedullary teratoma at L3 vertebral level were revealed in one case (Fig. 1) and tethered spinal cord with syringomyelia in the other (Fig. 2).

Patients were selected to be treated surgically if they met the following criteria: 1) the diameter of cyst is more than 1.5 cm;2) neurological symptoms and signs attributed to sacral perineural cysts that are serious enough to warrant treatment; 3) no or little response to medical and physical therapy. To determine whether the cyst is the culprit responsible for the symptoms, a trial of CT guided aspiration first.

Before 2006, six patients underwent sacral laminectomies, microsurgical cyst fenestration, and cyst wall imbrication with placement of free autologous fat or muscle grafts over the closed wall. Since 2006, seven patients underwent a modified surgical procedure. After sacral laminectomies, the cysts were fenestrated with a scalpel, for the draining of the fluid contents .The cyst wall was partially removed. After the procedure mentioned above, we would exam the aperture through which CSF may leak from the subarachnoid space to the cyst. The identified aperture was then repaired with a piece of fat and fibrin glue, for the prevention of CSF leakage. We observed that, in all cases (n = 6), the aperture was at the place through which nerve root traversed out of the subarachnoid space and into the cyst. In case that the aperture could not be identified (n = 1), the zone through which nerve root traversed the arachnoid was prophylactically covered with fat and fibrin glue. After careful hemostasis, the cyst cavity and local defect were covered with absorbable Gelfoam and fibrin glue. The wound was closed in water-tight layers. Postoperative lumbar drainage was not used in all of the seven patients because it was identified during the operation that CSF leakage stopped. In patients with additional pathological features (one patient with tethered spinal cord and a intramedullary teratoma in L3 vertebral level, the other patient with tethered spinal cord), these pathological entities were treated accordingly during the same operation procedure. Conservative management including the application of analgesic and non-steroid anti-inflammatory medication and physical therapy was administrated for two patients who rejected surgical treatment.

Patient outcomes were assessed by comparing the preoperative and postoperative examination results. The follow-up was conducted either by re-checking at outpatient clinic or by telephone questionnaires, with an averaged period as 40.1 months (range, 13 mo–10 yr ). All patients had MRI at greater than one year, and the averaged time of MRI for follow-up is 20.7 months.

## Results

**Figure 3:**
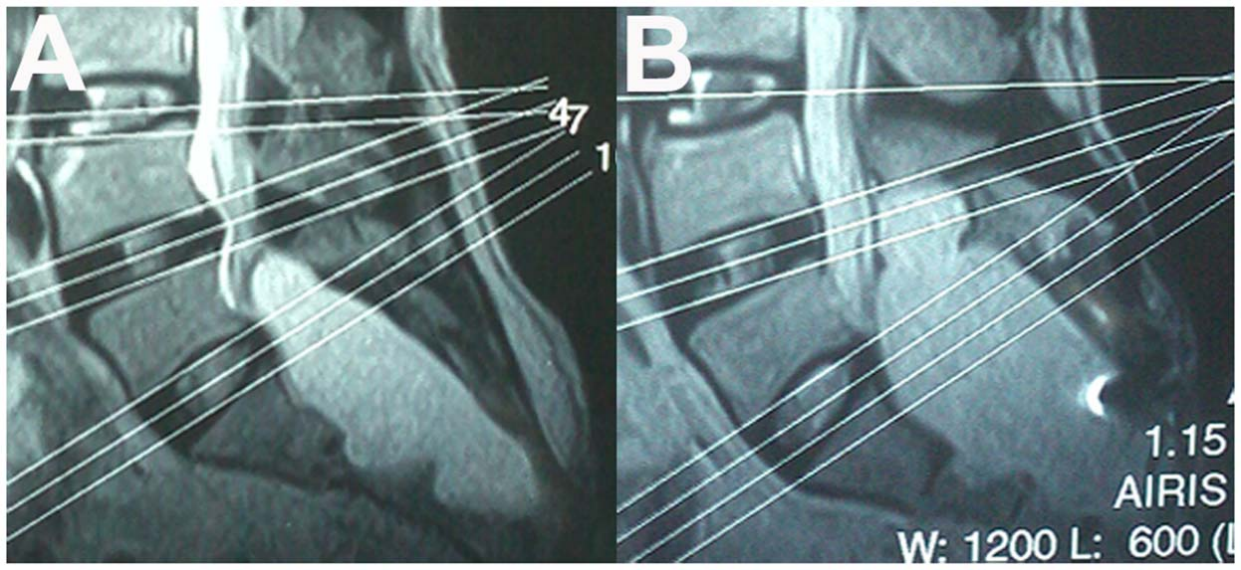
MRI study of recurrence of the cyst.A: Preoperative MRI showing a cyst in sacral spinal canal. B: Postoperative MRI showing the cyst reoccurring 8 months after the operation.

All of the six patients treated with microsurgical cyst fenestration and imbrication before 2006 experienced either complete or substantial resolution of their pre-operative symptoms and neurological deficits immediately after surgery or during follow-up visits (Table 1). However, there was one patient suffered form recurrent low back pain and bladder dysfunction eight months after the operation, which was subsequently confirmed by MRI study as the recurrence of the cyst (Fig. 3). This patient received second surgery thereafter, with no improvement of his symptoms. There was another patient who experienced CSF leakage, which was cured completely with an artificial dural patch in the second surgery and postoperative lumbar drainage for about one week.

Of the seven patients treated with the modified surgical procedure since 2006, six of them experienced complete or substantial relief of their preoperative symptoms and neurological deficits immediately after surgery or during follow-up visits (Table 1). There was only one patient whose preoperative bladder dysfunction was worsened after the operation. The patient’s condition recovered gradually to normal function one month later. Postoperative lumbar drainage for the prevention of CSF leakage was not administrated in all of the seven patients. There were no new postoperative neurological deficits, no CSF leaks, and no surgical infections. No recurrence was observed during the follow-up monitoring.

**Figure 4:**
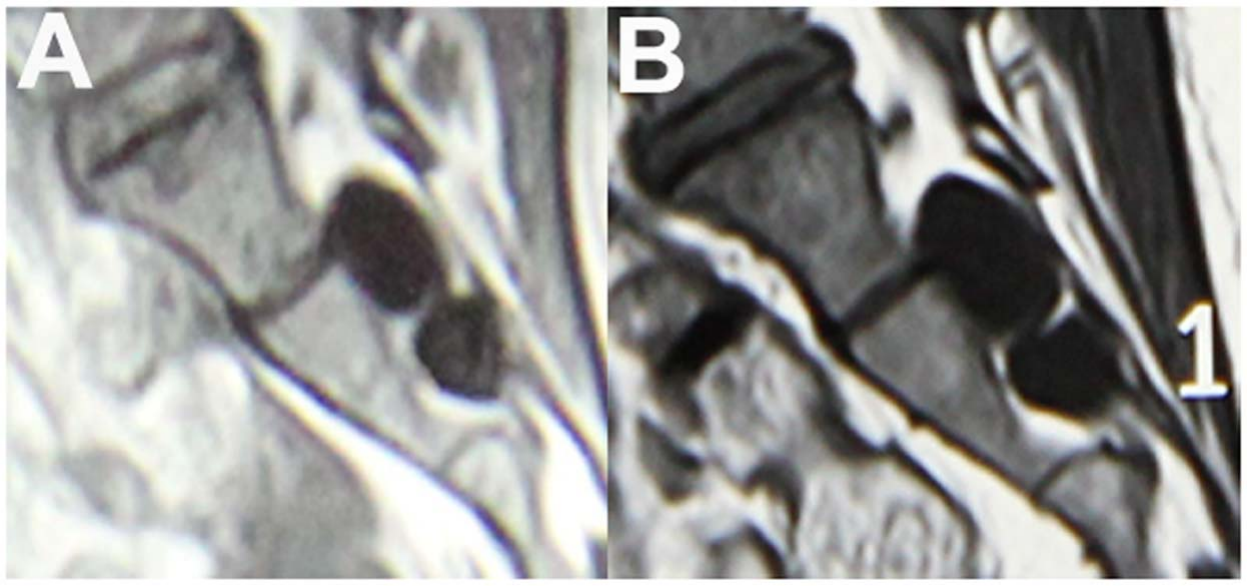
MRI study of progression of the cyst. A: MRI showing two cysts in sacral spinal canal. B: MRI showing that the cysts had grown.

The two patients treated with medication and physical therapy had no or little response to these conservative measures, and their symptoms aggravated with time. MRI examinations showed that the cysts in the two patients progress continuously. One patient’s cyst had grown 0.5 cm (from 1.9 to 2.4 cm) in diameter within 4 years(Fig. 4), the other’s increased 0.5 cm (from 1.5 to 2.0 cm )in diameter within 5 years.

## Discussion

The development of computed tomography (CT) myelography and MRI has led to an improvement in our ability to diagnose perineural cysts [2]. Despite advancements in diagnosis, there remains a great deal of controversy regarding the optimal treatment of symptomatic perineural cysts [2], [4], [5], [9]–[15].The reported treatment options include: 1) lumbar CSF drainage; 2) lumbar peritoneal shunt; 3) cyst subarachnoid shunt placement; 4) CT-guided percutaneous aspiration of the cyst with or without infusion of fibrin glue; 5) laminectomy for decompression of the cyst; 6) partial cyst removal and neck ligation with or without nerve root resection;7) partial cyst removal and cyst wall imbrication; 8) microsurgical cyst removal and cyst wall imbrication together with defect repairing with muscle, Gelfoam, or fibrin glue ; and 9) microsurgical fenestration of sacral perineural cysts to the thecal sac [2], [3], [5]–[11], [14], [16]. Those methods have been not satisfying because of variable rates of symptom resolution, cyst recurrence, as well as postoperative complications [2], [4]–[6], [8]–[11], [16].

Therefore, attempts to identify the aperture through which CSF may leak from the subarachnoid space to the cyst and repair it to stop CSF leak have been performed at our hospital since 2006. All of the seven patients treated with this surgical method obtained favorable results, although the aperture could not be located in one case. And there were no new postoperative neurological deficits, no CSF leaks, no surgical infections, and no recurrence. Postoperative lumbar drainage to prevent CSF leak was also not adopted. Based on our series,the procedure with partial cyst removal and the aperture repair for preventing CSF leakage from the subarachnoid space to the cyst appear to be more simple and effective.

In our series there were two patients with additional pathological features. One patient had tethered spinal cord and a intramedullary teratoma in L3 level, the other patient had tethered spinal cord with syringomyelia. Based on our knowledge, there have been no reports that sacral perineural cysts coexist with tethered spinal cord and intramedullary teratoma. It was difficult to evaluate which pathological entity caused the symptoms and neurological deficits in the two patients. We therefore treated surgically both perineural cysts and additional pathological entities during the same operation procedure.

In our series there were two patients who refused surgical treatment. During more than 4 years of follow-up monitoring, their cysts had grown significantly, and their symptoms developed worse with time, which may implicated the need of surgical intervention for symptomatic sacral perineural cysts.

In conclusion, it seems unlikely to conclude the optimal treatment based on single studies without significant number of patients, like this series presented here. More reported cases and further studies on management of sacral perineural cysts are needed. We recommend that microsurgical operation should be a treatment consideration and the method with partial cyst removal and the aperture repair for prevention of CSF leakage from the subarachnoid space seems to be more simple and effective.
